# Diseases of the Abdomen

**Published:** 1902-11-01

**Authors:** 


*(To be continued.)*

### Diseases of the Abdomen.

The construction of animal bioplasm, according to Harry Campbell,[1] involves a lavish expenditure of building material. Since the average annual increase of weight from birth to 20 is half stone, or less than one-third of an ounce a day, most of the food ingested is katabolised, which fact shows that katabolism must be very active at this period of life. The rate of katabolism, and hence the amount of food required, remains about the same from the age of maturity to 40. Then it progressively decreases until extreme old age is reached, when katabolism is so sluggish that little food is required. Men require more food than women as a whole, for katabolism is more rapid in men and anabolism in women. Idiosyncrasy depends to a large extent upon the activity of the thyroid gland.

Among the evils of over-eating must be placed anæmia,[2] which is due to the action of toxins upon the chromocytes. The action of overeating affects the respiration in more ways than one. It is embarrassed by obesity and an overloaded and distended stomach ; it is influenced by the stimulation of the splanchnic circulation, which excites respiratory action ; while the disturbance to the action of the heart and the hindrance to the passage of blood through the lungs does the same. Food not required by the organism has to be katabolised out of the way, with the resulting requirement of more oxygen and more complete expulsion of the $CO_2$ ; hence dyspnœa. Nature has made no provision against overeating ; food is absorbed from the digestive tract even though it may be worse than useless. It is oxidised and discharged with the production of heat, while store of useless energy leads to fits of irascibility or hysterical convulsions. The katabolised end products of excess of food are not so completely oxidised as with a mere sufficiency. The resulting waste is more toxic in its action. This toxæmia lowers the resistance to the action of microbes of inflammation to the toxins caused by diseased metabolism or by other poisons introduced into the body. Toxæmia accounts for the gastritis and enteritis of ill-fed children, for the delicate appearance of strumous children. Rickets may be a toxæmia of gastro-intestinal origin. Toxæmia is seen in gouty bronchitis, and suppuration is markedly affected by such toxic condition, and a recognised treatment of mammary abscess or a stye in the eye is a brisk purge.

The cardiac and arterial system are affected ; the kidneys try to relieve the toxæmia and the systemic arterioles contract to bring about a greater flow of blood through the kidneys, the result being a great strain upon the vessels and heart and degeneration of them. Over-eating hastens the fibroid encroachment of senility, the vascular system, liver, and kidneys suffering. Gout he holds to be a primary intoxication originating in the digestive viscera : errors of diet play an important part in its causation. Nervous disorders, headache, giddiness, tinnitus, irritability, drowsiness, etc. are caused by this toxæmia. In old age less and less food is required ; most of the people who have reached a great age have been moderate or small eaters. In these days of prepared foods there is a great danger of overfeeding infants.

Nature's safeguards against overfeeding comprise[3] vomiting, fasting, hæmorrhage, purgatives. Diet may be decreased by periodic fasting: certain elements may be left out or whole meals, such as breakfast, omitted. In dyspnœa especially a limit must be placed upon the quantity of food taken. Not only is there mechanical embarrassment of the heart and lungs, but there is indication of deficient oxygen and excess of $CO_2$. The excess of food demands the oxygen and gives out $CO_2$, and so makes matters worse. The diet must also be a non-flatulent diet.

Bronchitis calls for a spare diet, not only because excess tends to aggravate, but because it is prone to induce toxæmia, in itself a potent cause of bronchitis. This is particularly so in the bronchitis of past middle life. Asthma, morbus cordis, aneurysm, gout, Bright's disease, nervous affections, and febrile states all call for spare diet. Some states of indigestion, as well as cases of underweight, are benefited by decreasing the exercise and increasing the food.

According to Hutchison[4] the majority of the present day artificial foods do not warrant their existence. The requirements of such artificial food are that (1) it should stimulate the appetite ; (2) it should be easy to swallow and concentrated; but meat cannot be concentrated to more than 20 per cent. of its bulk without losing the proteid—sugar and oil are as concentrated carbohydrate and fat as it is possible to obtain. The need for peptonising foods is very much exaggerated. If the stomach is incapable of completing digestion the intestine will be able to do so, and unless the motor power of the stomach is deficient there will be no need to predigest the food. With regard to cost the patent preparations for the amount of units of energy are very much more expensive than ordinary foodstuffs. The foods made from milk, nutrose, eucasin, protene, plasmon, and casumen are all of value where it is impossible to give much food, and where it should be made as nutritious as possible. One special advantage is that milk and soups can be thickened with these without the patients being aware of their presence.

Cod-liver oil is of no more nutritive value, and is certainly dearer than milk or butter. Petroleum emulsion is never absorbed into the system, being recovered *in toto* from the stools. Pancreatic emulsion does not contain more fat than butter, and being made of lard is not more digestible than butter. Other foodstuffs contain fat and carbohydrates mixed (virol 20 per cent. fat, 6 per cent. carbohydrate, and virvis), and may be nutritious, but not more so than chocolate or Everton toffee. Bovril contains only the nutritive equivalent of 8 grammes of lean meat to one teaspoonful ; while the salts and extractives make it impossible to take much at a time.

With regard to meat juices, "Puro," a German product, shows the richest in proteid, but it is not all albumen of meat, some being egg albumen introduced. Brand's shows 15 per cent. albumen to 16 per cent. extractives, while Bovinine shows $\frac{1}{3}$ per cent. proteid and 3 per cent. extractives ; and whereas the majority give the spectrum of hæmoglobin, the latter shows that of methæmoglobin, and the question is whether it is not blood preserved in glycerine. In all the relative amount of foodstuff is very small. A meat juice can be easily manufactured at home at 1d. per ounce. The white of an egg with an equal quantity of water strained through muslin, and with any quantity of Liebig's extract one likes, dissolved in a little warm water, is most rich in coagulated albumin, and quite cheap. Peptone preparations cannot be given in sufficient quantities without producing diarrhœa. Somatose contains 80 per cent. of digested proteid (albumose, etc.).

With regard to the infant foods, the desiccated milk foods (Allenbury foods, Horlick's malted milk, Carnrick's soluble food, and Nestlé's food) are apt to be deficient in fat. Many of those foods in which the starch is to be digested in the process of making fail in this partially or completely. Mellin's food is good as an adjunct to milk : it contains no starch. The article further contains some valuable lists for comparison between various patent foods.

[1] Lancet, May 24. [2] Lancet, May 31. [3] Lancet, June 14. [4] Lancet, July 5.

